# Preparation and Characterization of Gluten/SDS/Chitosan Composite Hydrogel Based on Hydrophobic and Electrostatic Interactions

**DOI:** 10.3390/jfb14040222

**Published:** 2023-04-14

**Authors:** Guangfeng Li, Ni Lan, Yanling Huang, Chou Mo, Qiaoli Wang, Chaoxi Wu, Yifei Wang

**Affiliations:** 1 Department of Cell Biology, College of Life Science and Technology, Jinan University, Guangzhou 510642, China; 2 Guangdong Provincial Key Laboratory of Advanced Drug Delivery, Guangdong Provincial Engineering Center of Topical Precise Drug Delivery System, Guangdong Pharmaceutical University, Guangzhou 510006, China; 3 Key Laboratory of Innovative Technology Research on Natural Products and Cosmetics Raw Materials, Guangzhou 510642, China; 4 Guangdong Provincial Biotechnology Drug & Engineering Technology Research Center, Guangzhou 510642, China

**Keywords:** gluten, chitosan, electrostatic interaction, hydrophobic interaction, cytotoxicity

## Abstract

Gluten is a natural byproduct derived from wheat starch, possessing ideal biocompatibility. However, its poor mechanical properties and heterogeneous structure are not suitable for cell adhesion in biomedical applications. To resolve the issues, we prepare novel gluten (G)/sodium lauryl sulfate (SDS)/chitosan (CS) composite hydrogels by electrostatic and hydrophobic interactions. Specifically, gluten is modified by SDS to give it a negatively charged surface, and then it conjugates with positively charged chitosan to form the hydrogel. In addition, the composite formative process, surface morphology, secondary network structure, rheological property, thermal stability, and cytotoxicity are investigated. Moreover, this work demonstrates that the change can occur in surface hydrophobicity caused by the pH−eading influence of hydrogen bonds and polypeptide chains. Meanwhile, the reversible non−covalent bonding in the networks is beneficial to improving the stability of the hydrogels, which shows a prominent prospect in biomedical engineering.

## 1. Introduction

Recently, natural hydrogels with three−dimensional macromolecular networks have been applied to hemostatic wound dressings, electronic skins, and drug deliveries for their biocompatibility and biodegradability [1,2,3,4]. Specifically, gluten (G), a plant−derived protein extracted from wheat dough, is recognized as Generally Recognized as Safe (GRAS) by the U.S. Food and Drugs Administration (FDA) [5,6,7]. However, its poor mechanical properties limit its applicability [6,8,9]. On the other hand, chitosan (CS) exhibits a positively charged surface, which is endowed with antibacterial bioactivity for its widespread use in wound healing and tissue repair engineering [1,10,11].

Noticeably, people have devoted themselves to constructing strengthening networks through physical and covalent interactions, according to a previous report [12,13]. For instance, enhancing the stability of hydrogels has been achieved by preparing double interpenetrating, supramolecular bonding and hydrophobic−associated crosslinking networks [12,13,14]. Among them, hydrophobic interaction improves the toughness and dynamic durability of hydrogels without reducing water content [15,16]. Additionally, sodium lauryl sulfate (SDS) is an anionic and amphiphilic surfactant because of its non−polar hydrophobic tail (lipophilic) and a hydrophilic polar head (hydrophilic), which introduces biodegradability into SDS [17,18]. Moreover, SDS has electrostatic interaction with protein to expose its negatively charged surface [18]. It is assumed that SDS increases the water solubility and creates a group of negative charges of G, expecting to produce a new hydrogel by hydrophobic and electrostatic interactions [18,19,20]. After all, G is known to be water−insoluble [6]. To the best of our knowledge, no report has discovered SDS−modified gluten composite hydrogels. 

In this work, we fabricated SDS−modified G to give a negatively charged surface and then connected it with cationic CS by reversible non−covalent bonding. Meanwhile, the porous structure of G/SDS/CS composite hydrogels are beneficial for cell adhesion as biomedical materials due to hydrophobic interaction. Furthermore, several methods were carried out to confirm the physicochemical properties of the hydrogels, including mechanical property, thermal stability, and swelling ratio. As the electrostatic interaction forces change due to increasing pH, the particle size of hydrogel shows a trend of increasing and then decreasing. Especially, the cytotoxicity of the hydrogels were also evaluated for the consideration of being biomaterials. In general, this work prepared facile hydrogels, broadening the horizon to design composite hydrogels with superior properties.

## 2. Materials and Methods

### 2.1. Materials and Chemicals

Gluten (from wheat) (AR) and sodium lauryl sulfate (SDS) were purchased from Sigma−Aldrich. The chitosan (degree of deacetylation: 90%) was provided by Huan Tai County Jinhu Beach Shell Co., Ltd. (Shandong, China). A Cell Counting Kit−8 (CCK8) was purchased from Beyotime Biotechnology (Shanghai, China). Unless there were extra statements, all chemicals were of reagent grade and used as received, and all solutions were prepared with distilled water.

### 2.2. Preparation of the Hydrogel

The G/SDS/CS composite hydrogel was made using a modified version of a technique previously reported with some modifications [7]. At 25 °C, 0.12 g of SDS was first dispersed in 50 mL of ultrapure water and stirred for 0.5 h to completely dissolve. G was then dispersed in 50 mL of SDS solution with 0.5 h of stirring, and the concentration of G was 2 and 4 g per 100 mL of solvent (2% and 4% *w*/*v*). Subsequently, CS was mixed in aqueous acetic acid (2% *v*/*v*) to obtain solutions containing 2, 4, and 8 g CS per 100 mL solvent (2, 4, and 8% *w/v*). The solutions were stirred overnight (500 rpm for all stirring operations) and reserved for subsequent use. Furthermore, the G and CS solutions (5 mL each) were mixed at a ratio of 1:1 (*v*/*v*), and then the system was adjusted to pH 7 using small aliquots of either acid or base solution (1 M NaOH or 1 M HCl). After 1 h of stirring, it was incubated for 1 h at 60 °C and kept in an ice−water bath for 12 h to produce G/SDS/CS composite hydrogels. Finally, all were then dialyzed in distilled water for 72 h and lyophilized. All samples were pre-frozen at −80 °C for 24 h, then freeze−dried in the lyophilizer at −50 °C with a vacuum of 40 Pa. For later usage, the samples were kept at −20 °C. The G/CS hydrogels were prepared using the same procedure as the controls for comparison. The detailed composition of composite hydrogels is shown in Table 1.

### 2.3. Characterization of the Hydrogel

#### 2.3.1. Scanning Electron Microscope (SEM)

The microstructures of samples were examined using field emission scanning electron microscopy (FE−SEM). Freeze−dried composite hydrogels were transferred to the loading platform and coated with gold. After that, the microstructures were observed using an FE−SEM (ULTRA−55, ZEISS company, Oberkochen, Germany) with an accelerating voltage of 20 kV.

#### 2.3.2. Thermal Analysis

According to the methodology with some modifications [21], thermal gravimetric analysis−differential scanning calorimetry (TGA−DSC) (TGA/DSC3+/1600, Mettler Toledo, Hong Kong) was used to examine the thermal stability. Approximately 3 mg of freeze−dried hydrogels were weighed and sealed in an aluminum pan before being heated from 25 °C to 600 °C at a rate of 10 °C/min. Before testing, using a blank pan as a temperature calibrator, nitrogen was used as a purge gas at a rate of 50 mL/min.

#### 2.3.3. Dynamic Rheological Measurements

The rheological performance of the hydrogels was measured using conical plates on a Malvern Kinexus Pro+ rheometer at 25 °C.

(1) Frequency sweeps: The storage (G′) and loss (G″) moduli were determined by executing frequency sweeps with constant deformation and a strain amplitude of 0.5% over an angular frequency range of 0.1–100 rad/s (within the linear viscoelastic region).

(2) Shear thinning test: Under 0.5% strain, the model was utilized to test the viscosity properties of hydrogels with shear rates ranging from 0.1 to 100 s^−1^.

#### 2.3.4. Fourier Transform Infrared Spectroscopy (FTIR)

FTIR spectroscopy was used to investigate the secondary structures of samples, which were obtained using Nicolet IS50 + iN10 (Thermo Fisher Scientific, Waltham, MA, USA). FTIR analyses of samples were performed with a horizontal ATR Trough plate crystal cell in the 400–4000 cm^−1^ range, collecting automatic signal gains for 32 scans at room temperature at a resolution of 4 cm/s. The spectra were analyzed using the Omnic software package (version 6.1a, Thermo Nicolet Corp., Madison, WI, USA) and Peakfit software (version 4.12, SPSS Inc., Chicago, IL, USA).

#### 2.3.5. X-ray Diffraction (XRD)

The XRD pattern for each sample was determined using a polycrystalline X-ray apparatus (Bruker, Karlsruhe, Germany) at 40 kV and a current of 40 mA and Cu−Kα radiation with a wavelength of 0.2 nm. The scanning rate was 12°/min, and the diffraction angle (2θ) was varied from 4 to 40 degrees. 

#### 2.3.6. Measurement of Intrinsic Fluorescence Spectra

Based on the method [22], the intrinsic fluorescence spectra of the sample were obtained using an RF−6000 PC spectrofluorophotometer (Shimadzu Corp., Kyoto, Japan) at room temperature in quartz cuvettes. The sample solutions (0.2% *w*/*v*) were made in 0.05 M phosphate buffer (pH 7.5) and were then excited at 283 nm, and emission spectra were recorded in the 300–450 nm range. Both the emission and excitation slits were set to a wavelength of 5 nm.

#### 2.3.7. Measurement of UV Spectra

Sample solutions (0.2%, *w*/*v*) were dissolved using 0.05 M phosphate buffer (pH 7.5), centrifuged for 15 min at 8000× rpm, and then the supernatants were collected on a Techcomp UV1000 spectrophotometer (Shimadzu UV−1000, Kyoto, Japan) between 200 and 350 nm with 0.05 M phosphate buffer as the baseline. 

#### 2.3.8. Determination of Surface Hydrophobicity

The surface hydrophobicity of freeze−dried samples was determined using 8−anilino-1−naphthalenesulfonic acid (ANS) according to the method [22]. The samples were dissolved at 15 mg/mL using 0.01 M phosphate buffer (pH 7.0) and agitated for 2 h. The solution was centrifuged for 10 min at 8000× rpm, and the supernatants were then diluted in a series of gradients. Subsequently, ANS solutions were diluted at 8 mM using 0.1 M phosphate buffer and incubated for 15 min, and then the fluorescence intensity of the solvent was measured using a fluorescence spectrophotometer (Shimadzu Corp., Kyoto, Japan) with the excitation and emission wavelengths of 390 and 470 nm, respectively. The gradient of fluorescence intensity with protein concentration was associated with the surface hydrophobicity of samples.

#### 2.3.9. Particle Size and Zeta-Potential Analysis

After dilution with double−distilled water to an appropriate concentration, the particle size, polydispersity index (PDI), and zeta potential were evaluated using a Nano-ZS90 zeta−plus (Malvern Zetasizer Nano ZS, Malvern, UK) based on dynamic light scattering (DLS). The experiment was carried out in triplicate.

#### 2.3.10. Swelling Behavior of the Hydrogels

Hydrogel swelling experiments were gravimetrically examined by evaluating their water uptake capacity using the method [23]. The dry hydrogels were weighed before being stored in phosphate buffer solutions of pH 7.4 at 25 °C. After immersing for a certain amount of time, hydrogels were wiped with tissue paper to remove the water from the surface. The amount of water absorbed by hydrogels was calculated using the equation below:

swelling percentage (%) = [(W_s_ − W_d_)/W_d_] × 100%

W_d_ and W_s_ are the weights of hydrogels when dry and swollen, respectively.

#### 2.3.11. In Vitro Cytotoxicity

The cytotoxicity of hydrogels was determined using a Cell Counting Kit−8 (CCK8) assay using HUVEC, BALB/c−3T3 fibroblasts, and human keratinocytes (HaCaT cells) (from CAS Shanghai Cell Bank, Shanghai, China). Briefly, the samples (1 mg/mL) were extracted for 72 h using a cell culture medium at 37 °C. The prepared hydrogels were ultrasonically sonicated for about 1 h at high speed and passed through a 0.22 μm filter, and then cells were treated with them. The cells were planted in 96−well plates (1.0 × 10^4^/well) and incubated for 24 h, after which they were incubated with a range of concentrations of the hydrogel solutions for 24 h at 37 °C in a humidified atmosphere with 5% CO_2_. The CCK8 solution was then added to each well, followed by a further 2.5 h incubation. Absorbance in each well was then assessed at 450 nm. All processes were repeated three times, with fresh culture medium cells acting as a negative control.

#### 2.3.12. Statistical Analysis

The experimental data were presented in triplicates as mean ± standard deviation. The analysis of variance (ANOVA) was utilized to statistically analyze the results, and the differences of *p* < 0.05 were judged significant in all situations.

## 3. Results and Discussion

### 3.1. Preparation of the Hydrogel

**Figure 1:**
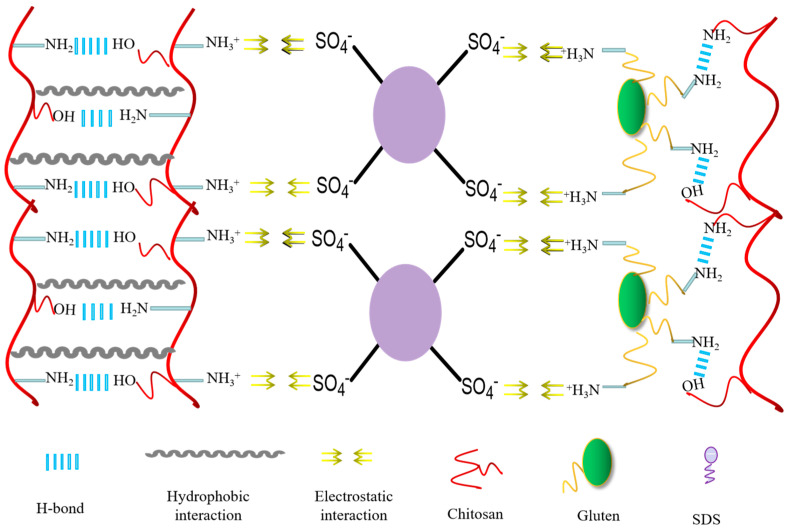
The cross−linking schematic of G/SDS/CS composite hydrogels.

In the experiments, G/SDS/CS composite hydrogels were synthesized by mixing aqueous solutions of modified G and CS with prolonged agitation to prevent chitosan particles from settling, with immediate cross−linking and freeze−drying afterwards. The cross−linking schematic representation of the composite hydrogel is shown in Figure 1. In contrast to the composite hydrogel, the G/CS hydrogel is not structurally stable and cannot meet the fundamental mechanical testing requirements. Compared to other composite hydrogels, the hydrogel defined as G_4_SDS_0,83_CS_4_ exhibited better stability for subsequent experiments.

### 3.2. Characterization of the Hydrogel

#### 3.2.1. Scanning Electron Microscope (SEM)

**Figure 2:**
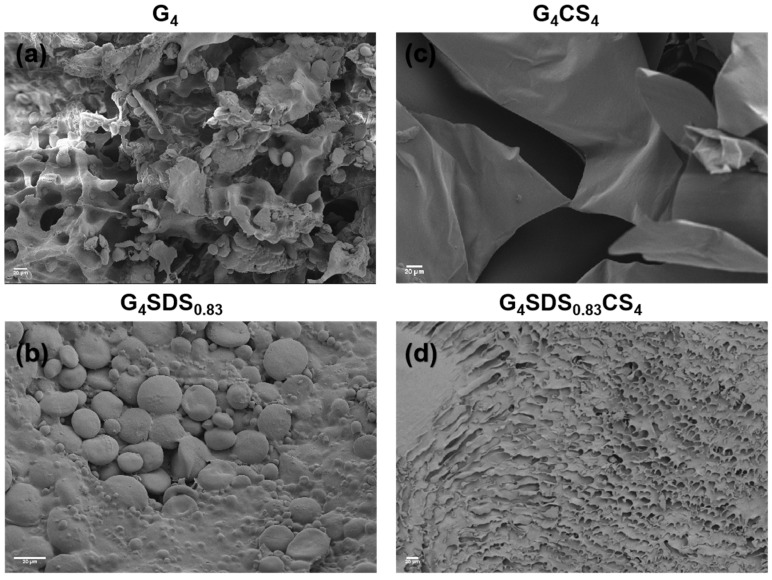
SEM images of G_4_ (**a**)_,_ G_4_SDS (**b**), G_4_CS_4_ (**c**), and G_4_SDSCS_4_ (**d**) hydrogels, Scale bar: 20 µm.

In the freeze−drying process, the hydrogel rapidly freezes at low temperature. Then, the water in the internal space immediately turns to ice and sublimates in a vacuum to preserve the internal structure of the hydrogel, the pore size of which when formed is strongly related to the original ice crystals and a variety of factors [24]. The SEM micrographs of the freeze−dried hydrogels are shown in Figure 2. G was a globular protein with a granular appearance (Figure 2a). The neat G was not homogeneous in size and shape, indicating that G is aggregated and has little active site exposure [25]. The morphology of G modified by SDS dramatically changed, exhibiting a coarse surface morphology with particles that were not uniform in size and shape (Figure 2b), which may be a result of the exposure to hydrophobic amino acids, and thus, free NH_2_ and OH groups on the G backbone. 

In addition, G/CS hydrogels had an irregular and incomplete structure with multiple layers of lamellae (Figure 2c), which promoted adhesion but did not facilitate further applications due to the lack of reticulation structure [7,26]. In the G_4_SDS_0.83_CS_4_ composite hydrogels (Figure 2d), internal pores were regularly distributed with a porosity of 32.3 ± 1%, and their cross−linking density varied according to the content of G or CS [22,25,27]. Compared to the G_4_CS_4_ hydrogels, G_4_SDS_0.83_CS_4_ composite hydrogels with homogenous pores on the surface and within the cavities can promote cell adhesion and proliferation [28]. This three−dimensional mesh of interconnected pore structures indicates the potential of the hydrogels for drug delivery applications.

#### 3.2.2. Thermal Analysis

**Figure 3:**
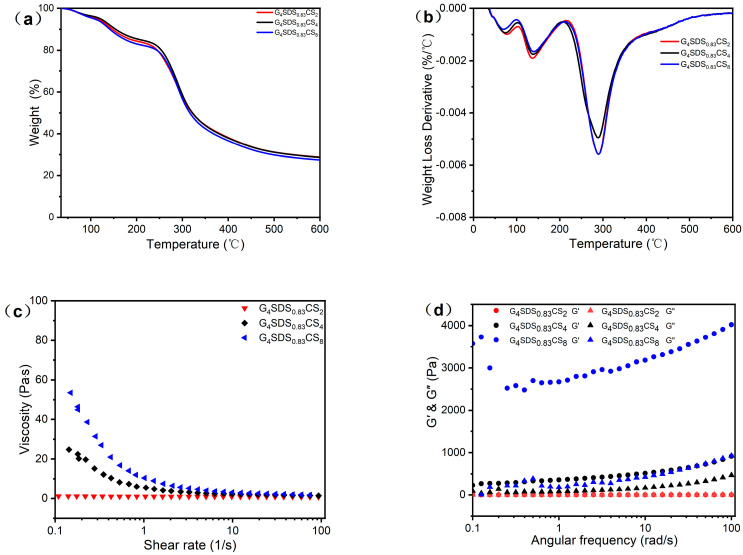
Thermal properties of G_4_SDS_0.83_CS_2_, G_4_SDS_0.83_CS_4_, and G_4_SDS_0.83_CS_8_ hydrogels: (**a**) TGA thermograms, (**b**) DTG thermograms. Rheological studies of G_4_SDS_0.83_CS_2_, G_4_SDS_0.83_CS_4_, and G_4_SDS_0.83_CS_8_ hydrogels: (**c**) Shear thinning test showing the relationship of viscosity with the shear rate at 25 °C, (**d**) Frequency sweep test displaying the G′/G″ values.

TGA was carried out to investigate the degradation temperature of materials, as shown in Figure 3a. Figure 3b depicts derivative thermogravimetric (DTG) curves for TGA. As demonstrated in Table 2, the weight loss of G mostly occurred between 40 °C and 350 °C, with two major stages of mass loss, with degradation temperatures and residual mass rates of 313.67 °C and 25.10%, respectively. The first weight loss between 40 °C and 100 °C was attributed to hydrogen bond breakdown and decomposition, and the loss of free and bonded water [22]. The second weight loss was chiefly caused by the breakdown of covalent peptide bonds between amino acid residues, as well as the breakdown of S−S, O−N, and O−O bonds amongst protein molecules [22,29]. Compared to G_4_SDS_0.83_, the degradation temperature of G_4_SDS_0.83_CS_4_ increased from 281.86 °C to 290.23 °C, respectively, and the residual weight increased from 29.78% to 30.29%, indicating various interactions between the three components and a more stable structure. The weight loss in the second phase differed with the addition of SDS, which could be attributed to the diverse network architectures generated.

Furthermore, the influence of CS concentration on the thermal stability of the hydrogels was investigated. As the concentration grew, the maximum degradation temperature fell, while the weight loss increased [22,23,30]. It is suggested that a high CS concentration may be deleterious to creating a stable G protein hydrogel structure with a bigger pore size, a stiffer texture, and a sparser product structure. This conclusion is supported by the DSC data (Figure S1). Therefore, the hydrogel defined as G_4_SDS_0.83_CS_4_ was used in subsequent experiments due to its high retention rate and the advantage of a corresponding temperature at 10% weight loss. All these results show that the modified G forms a thermally stable system with the crosslinked structure of CS, which improves the stability of the hydrogel.

#### 3.2.3. Dynamic Rheological Measurements

A dynamic shear rheometer was used to assess the viscoelastic characteristics of the hydrogels. Figure 3c shows the variation of viscosity with a shear rate of G_4_SDS_0.83_CS_4_ at room temperature. All solutions displayed non−Newtonian shear thinning behavior, with a rising change in viscosity with increasing concentration, indicating these hydrogels are entanglement−rich by the electrostatic interaction between CS and modified G [23,30]. In particular, this implies that these hydrogels have excellent shear−thinning capabilities, which are essential for injectability. This result may be explained by the fact that the structure of SDS contains a higher viscous density, limiting the chain mobility in the hydrogel matrix and resulting in a stiff network [7,27]. According to Figure 3d, all the hydrogels displayed mechanical robustness, with storage modulus G′ values greater than loss modulus G″ values over the entire experimental angular frequency range. Moreover, all of the G′ and G″ of hydrogels steadily increased with frequency, demonstrating that the hydrogels are both elastic and solid [22,30]. The result showed that the interactions between modified G and CS in solution produced a gel−like structure. The G_4_SDS_0.83_CS_4_ hydrogel had high viscosities, with high concentrations of CS enhancing its viscoelastic characteristics [23,30].

#### 3.2.4. Fourier Transform Infrared Spectroscopy (FTIR)

**Figure 4:**
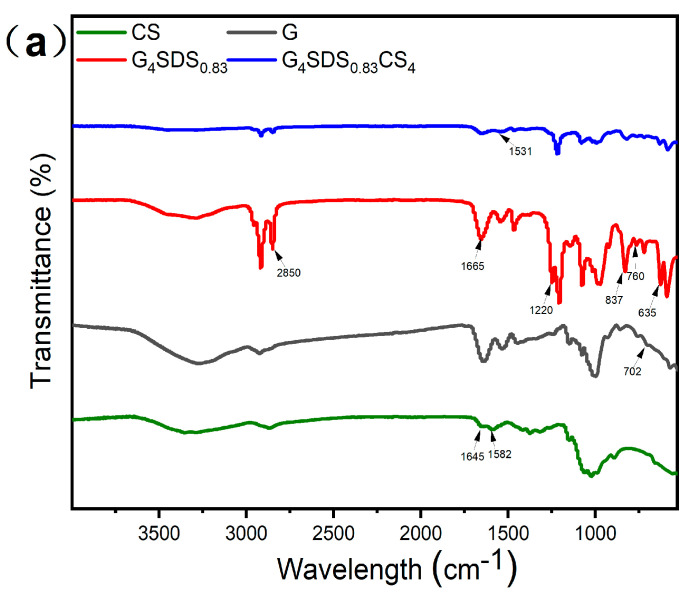

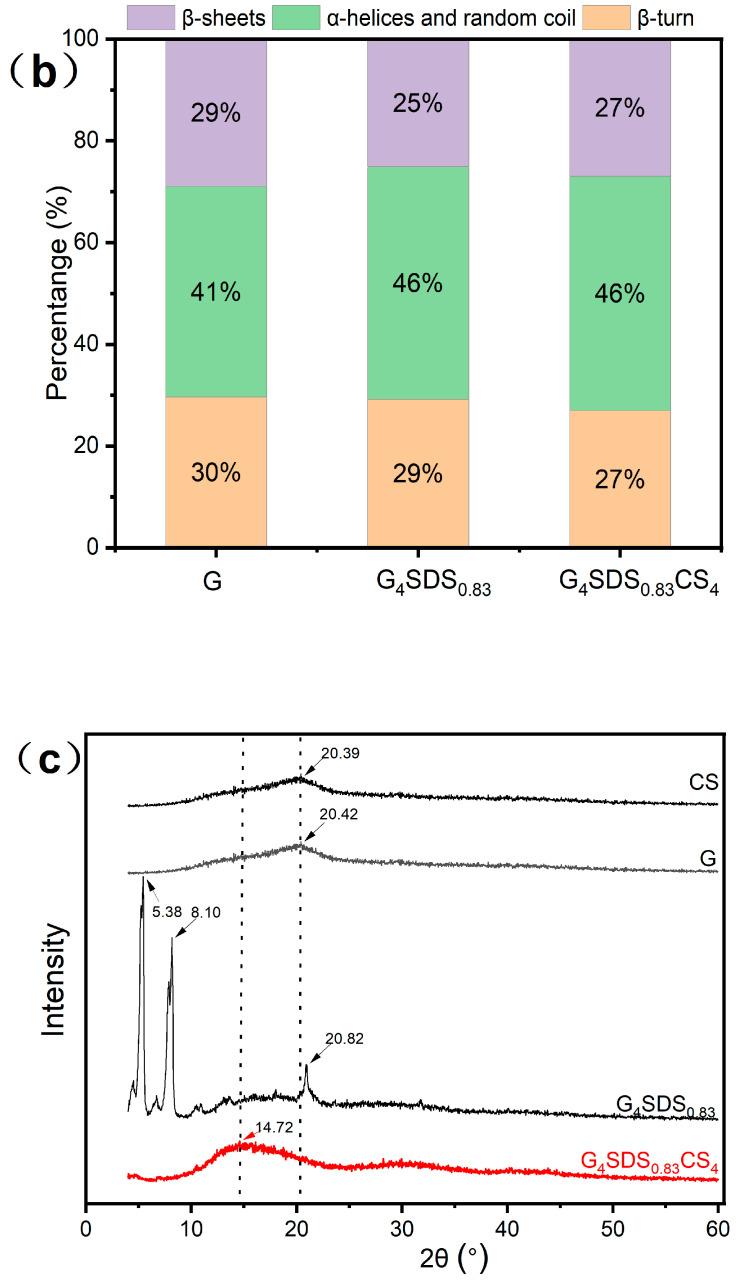
(**a**) FITR spectra of G, CS, G_4_SDS_0.83_, and G_4_SDS_0.83_CS_4_ samples; (**b**) The relative proportion of secondary structure (1615−1700 cm^−1^); (**c**) XRD spectra of G, CS, G_4_SDS_0.83_, and G_4_SDS_0.83_CS_4_ samples.

The ATR−FTIR spectra provide more insight into the structural properties of the produced hydrogel scaffolds. The chemical structures of G, CS, G_4_SDS_0.83_, and G_4_SDS_0.83_CS_4_ samples are shown in Figure 4a. The characteristic signals of SDS were 1665 and 1220 cm^−1^ (SO_2_ stretching); 837 and 760 cm^−1^ (asymmetric and symmetric stretching of S−O−C); 635 cm^−1^ (SO_3_ bending) [31,32]. Furthermore, we discovered a distinct peak of G at 702 cm^−1^ in all gluten−containing samples [25]. Meanwhile, all chitosan−containing samples displayed a distinct absorption peak at 1645 cm^−1^, attributed to the C=O stretching vibration (amide I) of chitosan’s N−acetyl groups [31,33]. The peak at 1582 cm^−1^ for CS was related to the N−H bending vibration of the primary amine group. Figure 4a shows a shift in this peak from 1582 cm^−1^ to 1527 cm^−1^ for the G_4_SDS_0.83_CS_4_ sample, showing a reduction in the N−H bending vibration of the primary amine. The presence of a conspicuous electrostatic interaction is shown by this peak shift [31,34].

Based on the unique infrared absorption band of specific functional groups, FTIR spectroscopy is frequently employed to examine the biopolymer’s secondary structure and conformation [35]. The bands located at 1615−1640 cm^−1^ and 1690−1700 cm^−1^ are assigned to the β−sheets, whereas 1640−1660 cm^−1^ is assigned to the α−helix and random coil, and the β−turn is allocated areas of 1660−1690 cm^−1^, correspondingly [36]. Figure 4b shows that the a−helical and random curl content rose following SDS modification, whereas the β−sheets decreased and the β−turns declined. The β−sheets increased while the β−turns decreased after cross−linking with CS. This result shows that the β−turns are converted to β−sheets during the gelling process with CS, while the α−helix and random coil remain unchanged, as can be deduced. The decrease in the a−helix/β−sheet content ratio indicates that G adopted a more stable structure with the addition of CS [37].

#### 3.2.5. X-ray Diffraction (XRD)

X-ray diffraction analysis was used to investigate the crystal structure of G, CS, G_4_SDS_0.83_, and G_4_SDS_0.83_CS_4_ complexes. Figure 4c showed that no significant diffraction peaks were discovered in the spectra of G, which was due to its amorphous state [38]. When G was modified by SDS, the crystallinity of G increased from 5.63% to 11.8%, with the XRD pattern revealing several sharp and strong peaks with the characteristic diffraction peaks of SDS [23,39]. These peaks vanished when G_4_SDS_0.83_ was complexed with CS to form a complex. In brief, the spectra revealed that the G_4_SDS_0.83_CS_4_ complex was amorphous, indicating that the complexation process had minimal effect on the state of G [27,38]. This result indicated that SDS broke the structure of G and caused its molecules to unfold, allowing crystalline areas to form during the binding process. In general, crystallinity decreases as the degree of cross−linking increases. The addition of CS reduced the crystallinity of the complex from 11.8% to 2.6%, showing that the reaction with CS increased cross−linking. It is attributable to forming polyfunctional groups and long chains, which produce a more compact network system [1,38,39]. All such results indicate that the strong cross−linking between G_4_SDS_0.83_ and CS reduces the hydrogen bonding and leads to the formation of amorphous structures in the hydrogel.

#### 3.2.6. Measurement of Intrinsic Fluorescence Spectra

**Figure 5:**
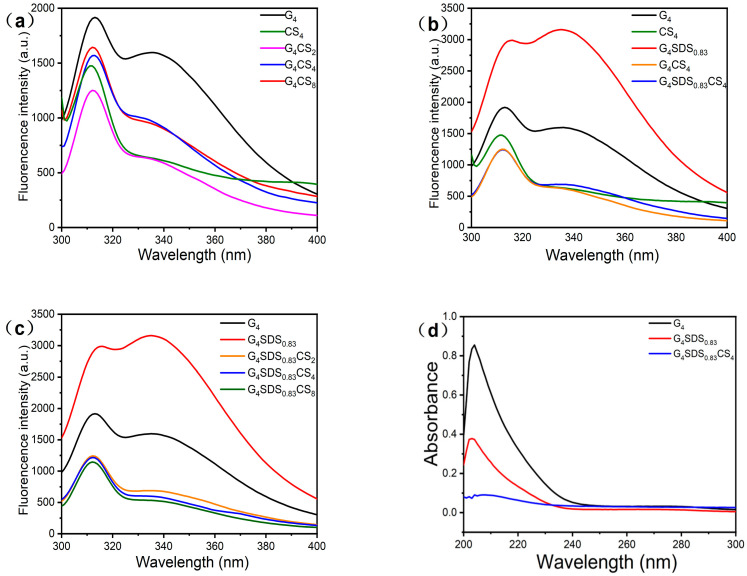
(**a**) Intrinsic fluorescence of G_4,_ CS_4,_ G_4_CS_2,_ G_4_CS_4,_ and G_4_CS_8_ samples; (**b**) Intrinsic fluorescence of G_4_, CS_4,_ G_4_SDS_0.83_,G_4_CS_4_ and G_4_SDS_0.83_CS_4_ samples; (**c**) Intrinsic fluorescence of G_4_, G_4_SDS_0.83_, G_4_SDS_0.83_CS_2,_G_4_SDS_0.83_CS_4_ and G_4_SDS_0.83_CS_4_ samples; (**d**) UV spectra of G_4_, G_4_SDS_0.83_ and G_4_SDS_0.83_CS_4_ samples.

Intrinsic fluorescence analysis was further employed to investigate the intermolecular interactions in the G hydrogel and validate the conformational alterations. As shown in Figure 5a, the maximum fluorescence emission of natural G was approximately 311 nm at an excitation wavelength of 283 nm, in addition to a fluorescence absorption peak at 340 nm, which was attributed to the fluorescence peaks of Trp residues [22]. The fluorescence intensity at 340 nm was dramatically reduced when G and CS were bound, implying that the complexation of G with CS may lead to the quenching of the fluorescence of G. Moreover, based on the fluorescence quenching result, we also investigated the changes in the fluorescence intensity of the polymer with increasing CS concentration. As a result, the increase in fluorescence intensity of the polymer may be attributed to CS fluorescence absorption [40,41,42].

In addition, as shown in Figure 5b, the modifier’s effect on fluorescence was investigated. The inclusion of the SDS alteration resulted in a slight red shift of the G and an increase in fluorescence intensity, which was attributed to the modifier exposing the hydrophobic peptide chain. The migration of hydrophobic amino acids into a polar environment revealed that the protein structure had relaxed due to the modification, exposing hidden residues such as Trp. When the modified G was combined with CS, the fluorescence intensity sharply dropped, indicating that the microenvironment had turned hydrophobic. The reason for this is the electrostatic attraction between the negatively charged G and the positively charged CS, as well as the fact that SDS molecules can synergistically bind to CS substrates, creating an enthalpy change and therefore facilitating the collision of CS with individual SDS and G molecules [29,42]. Based on increasing the concentration of CS under the same conditions as modified G and observing the change in fluorescence intensity in Figure 5c, we observed a decrease in fluorescence intensity as the concentration of CS increased, which was attributed to the reaction and masking of hydrophobic amino acids by CS [22,29]. The fluorescence spectra results are in agreement with the UV spectra analysis.

#### 3.2.7. Measurement of UV Spectra

The UV spectra are used to identify changes in the chromophores (aromatic amino acid side chains), which shed light on the protein structure [40]. The UV spectra of G at various phases are shown in Figure 5d. The UV spectra of G displayed typical peaks of about 205 and 270 nm, corresponding to peptide bonds and aromatic amino acid residues (phenylalanine, tyrosine, and tryptophan, respectively) [41]. After SDS modification, the absorption peaks of aromatic amino acids were lower, indicating a change in the chromophore’s microenvironment. The minor blue shift in the absorption peak of the aromatic amino acids is explained by the SDS exposing the hydrophobic amino acids in G to a more polar environment. In addition, the considerable drop in absorbance is due to the creation of a high molecular weight polymer, which produces a “steric hindrance” effect that transforms its conformation from a planar to a non−planar state, partially masking the non-polar aromatic side chain residues [40,41]. 

Moreover, as shown in Figure S2, we also investigated how pH affected the UV absorbance of the compounds. The literature reports isoelectric point (pI) values of 6.12 for G and 7.8 for CS [6,25]. We discovered that the lowest absorbance was recorded as close to pH 7.0, which was most probably related to the greatest stability of polymer complexes. These results indicate the successful synthesis of G_4_SDS_0.83_CS_4_ hydrogels

#### 3.2.8. Determination of Surface Hydrophobicity

**Figure 6:**
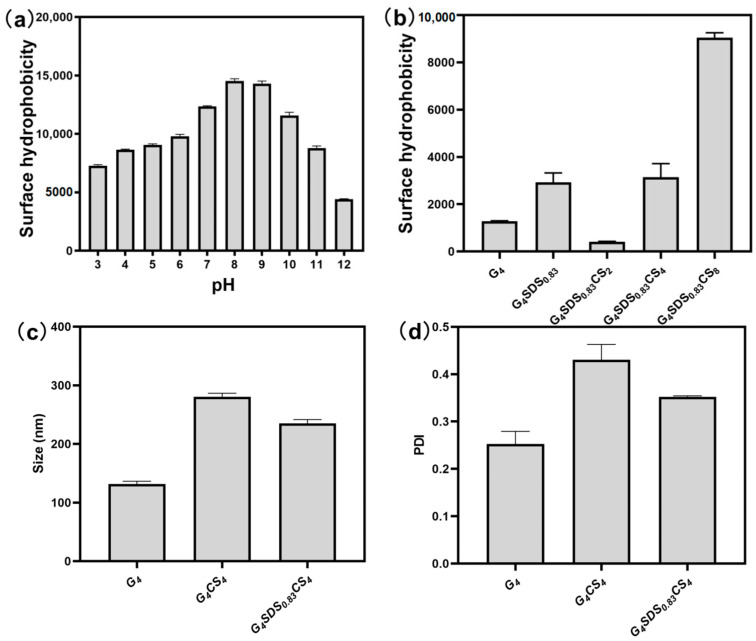
(**a**) The surface hydrophobicity of G_4_SDS_0.83_CS_4_ sample of different pH values; (**b**) The surface hydrophobicity of different phases; (**c**) Size of G_4_, G_4_SDS_0.83_, and G_4_SDS_0.83_CS_4_ samples; (**d**) PDI of G_4_, G_4_SDS_0.83_, and G_4_SDS_0.83_CS_4_ samples.

As shown in Figure 6a, the effect of pH on the surface hydrophobicity of the complexes was examined. The surface hydrophobicity increases from pH 3.0 to 9.0 and then declines from pH 9.0 to 12.0. At pH 3.0–9.0, it is suggested that the positively charged CS and negatively charged modified G are tightly bound due to electrostatic interactions. Therefore, the corresponding binding sites for ANS and G become fewer, and surface hydrophobicity is low. However, a rise in pH reduces the binding intensity of composite hydrogel, increases the binding sites for ANS, and promotes surface hydrophobicity [22,43]. As the pH increases from 9.0 to 12.0, G and CS become the same charge. In response to increased pH, electrostatic repulsion causes G to become tightly bound, leading to fewer related binding sites for ANS and reduced surface hydrophobicity. As demonstrated in Figure 6b, the hydrophobicity of G was reduced after modification with SDS and mixing with CS [43]. Furthermore, as the CS concentration increased while the protein concentration remained constant, the surface hydrophobicity of the complex increased, which may be a result of CS’s intrinsic surface hydrophobicity.

#### 3.2.9. Particle Size and Zeta-Potential Analysis

Particle size and PDI changes during the complex formation phase are shown in Figure 6c,d. G particles were approximately 131.6 nm in size before being increased to approximately 280.6 nm by SDS modification. Then, the particle size decreased to 235.0 nm when complexed with CS. Briefly, particle size was increased due to a disorder in G’s spatial structure caused by SDS [44,45]. When CS was introduced to the complex, the electrostatic attraction between the modified G and CS aided in creating the complex, which became denser and had smaller particles [7,38]. In addition, to clarify the molecules’ interaction, G and CS’s zeta potentials were determined in the pH range of 3.0–12.0, as shown in Figure S3. The zeta potential of G in the above pH range was less than zero and fluctuated, indicating that the modified G contains a negative charge. Meanwhile, the zeta potential of CS steadily decreased with increasing pH in the pH range of 3.0–12.0. The potential of CS was zero at a pH of 8.8. Moreover, the absolute potential difference value decreases in the pH 3.0–9.0 range and increases in the pH 9.0–12.0 range, which is attributed to electrostatic interactions, mainly electrostatic attraction at pH 3.0–9.0 and electrostatic repulsion at pH 9.0–12.0 [44]. This result is consistent with the trend toward surface hydrophobicity.

#### 3.2.10. Swelling Behavior of the Hydrogels

**Figure 7:**
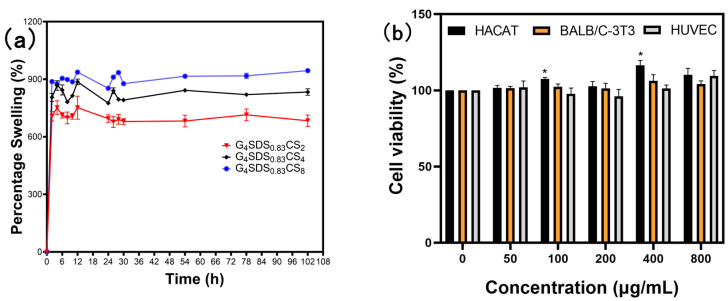
(**a**) Percentage swelling plot of G_4_SDS_0.83_CS, G_4_SDS_0.83_CS_4_, and G_4_SDS_0.83_CS_8_ hydrogels at 25 °C; (**b**) The cell viability of G_4_SDS_0.83_CS_4_ hydrogels to HaCaT, BALB/c-3T3, and HUVEC cells of various concentrations. (n = 3,* *p* < 0.05 vs. the corresponding negative control group).

As shown in Figure 7a, the swelling behavior of hydrogels was examined at room temperature in PBS solutions of pH 7.4. The swelling behavior reached equilibrium after 12 h. There was capillary absorption in the interior structure of the hydrogel at the start of swelling. In the three−dimensional network, the porous structure offers routes for molecules to enter and exit. To form the hydration layer, hydrophilic groups (−OH, −COOH, and −COO−) are bound to water molecules via ligands or hydrogen bonds [46]. The swelling characteristics of the hydrogel are mainly determined by the CS concentration. A possible explanation for this might be that both CS and G were involved in creating the hydrogel crosslinking network. On the one hand, the dense network structure of the hydrogel formed by the higher G content limits the mobility of the polymer chains, resulting in reduced interaction with water molecules. On the other hand, the amine and hydroxyl groups of CS easily hydrate with water due to their hydrophilicity. As a result, the ability of the hydrogel to form hydrogen bonds with water molecules increases, making swelling more visible [23,46].

#### 3.2.11. In Vitro Cytotoxicity

The biocompatibility of hydrogels is critical for its application as biomedical materials [47,48]. After 24 h of incubation, the CCK8 assay confirmed the non-cytotoxicity of G_4_SDS_0.82_G_4_ (50, 100, 200, 400, and 800 μg/mL) to BALB/c−3T3 and HUVEC. As shown in Figure 7b, the cell viability was up to 100%, and there were no statistically significant differences compared to the controls. Furthermore, the cell viability of HaCaT cells mediated by G_4_SDS_0.82_G_4_ exhibited statistically significant variations (*p* < 0.05), indicating that it may have the ability to enhance cell proliferation at specific concentrations. This finding reveals that these hydrogels are very biocompatible. It was demonstrated that these hydrogels have potential use in medications, medical devices, and biomedical materials [49,50].

## 4. Conclusions

In summary, we prepared gluten G/SDS/CS (gluten/sodium lauryl sulfate/chitosan) composite hydrogels by combining SDS−modified G and CS with a porous structure, which was beneficial to cell adhesion in biomedical applications. Furthermore, the negative surface of G caused by SDS strongly connected with positively charged CS through electrostatic and hydrophobic interactions, and thus the mechanical performance of the hydrogels was effectively improved. Meanwhile, the change in pH values also contributed to enhance the stability of the hydrogels by promoting the binding of proteins and polysaccharides. Moreover, several methods were carried out to study the self-healing property, injectability, and thermal stability of the hydrogels. Specifically, the composite hydrogels exhibited no cytotoxicity against the studied cell lines, which opens the scope of the composite hydrogels as being used as biomaterials.

## Figures and Tables

**Table 1:** Composition of G/SDS/CS and G/CS hydrogels.

Abbreviation	G (*w*/*v*) ^a^	SDS (mM)	CS (*v*/*v*) ^a^
G_2_SDS_0.83_CS_2_	2%	8.3	2%
G_2_SDS_0.83_CS_4_	2%	8.3	4%
G_2_SDS_0.83_CS_8_	2%	8.3	8%
G_4_SDS_0.83_CS_2_	4%	8.3	2%
G_4_SDS_0.83_CS_4_	4%	8.3	4%
G_4_SDS_0.83_CS_8_	4%	8.3	8%
G_4_	4%	0%	0%
G_4_SDS_0.83_	4%	8.3	0%
CS_4_	0%	0%	4%
G_2_CS_2_	2%	0%	2%
G_2_CS_4_	2%	0%	4%
G_2_CS_8_	2%	0%	8%
G_4_CS_2_	4%	0%	2%
G_4_CS_4_	4%	0%	4%
G_4_CS_8_	4%	0%	8%

^a^ Concentration before mixture.

**Table 2:** The TGA results of different phases of samples.

Samples	T_10%_ ^a^ (°C)	T_30%_ ^a^ (°C)	T_50%_^a^ (°C)	Td ^b^ (°C)	Residual Mass (wt % )
G_4_	276	306	329	308	25.10
G_4_SDS_0.83_	255	290	343	281	29.77
G_4_CS_4_	176	284	353	292	41.41
G_4_SDS_0.83_CS_2_	147	274	321	290	30.15
G_4_SDS_0.83_CS_4_	154	279	322	289	30.29
G_4_SDS_0.83_CS_8_	142	278	316	288	28.52

^a^ Temperature corresponding to 10, 30, and 50% weight loss, respectively. ^b^ Temperature at the maximum degradation rate.

## Data Availability

Research data are not shared.

## Supplementary Materials

The following supporting information can be downloaded at: https://www.mdpi.com/article/10.3390/jfb14040222/s1, Figure S1: DSC thermograms.of G_4_SDS_0.83_CS_2,_ G_4_SDS_0.83_CS_4,_ and G_4_SDS_0.83_CS_8_ samples; Figure S2: UV spectra of G_4_SDS_0.83_CS_4_ sample at different pH; Figure S3: Zeta potential and the absolute potential value of G and CS at different pH.
