# Supplementary material for: Preparation and Characterization of Gluten/SDS/Chitosan Composite Hydrogel Based on Hydrophobic and Electrostatic Interactions
Source: J Funct Biomater. 2023 Apr 14;14(4):222. doi: 10.3390/jfb14040222 (PMC10146719; doi:10.3390/jfb14040222)
Supplement: Supplementary file 1 [file jfb-14-00222-s001.zip › jfb-2308478-supplementary.pdf]

Supplementary Materials

Preparation and Characterization of Gluten/SDS/Chitosan Composite Hydrogel Based on Hydrophobic and Electrostatic Interactions

Guangfeng Li [1], Ni Lan [1], Yanling Huang [1], Chou Mo [2], Qiaoli Wang [1], Chaoxi Wu [1,3,*] and Yifei Wang [1,3,4,*]

[1] Department of Cell Biology, College of Life Science and Technology, Jinan University, Guangzhou 510642, China
[2] Guangdong Provincial Key Laboratory of Advanced Drug Delivery, Guangdong Provincial Engineering Center of Topical Precise Drug Delivery System, Guangdong Pharmaceutical University, Guangzhou 510006, China
[3] Key Laboratory of Innovative Technology Research on Natural Products and Cosmetics Raw Materials, Guangzhou 510642, China
[4] Guangdong Provincial Biotechnology Drug & Engineering Technology Research Center, Guangzhou 510642, China
* Correspondence: chaoxiw@gmail.com (C.W.); twang-yf@163.com (Y.W.)

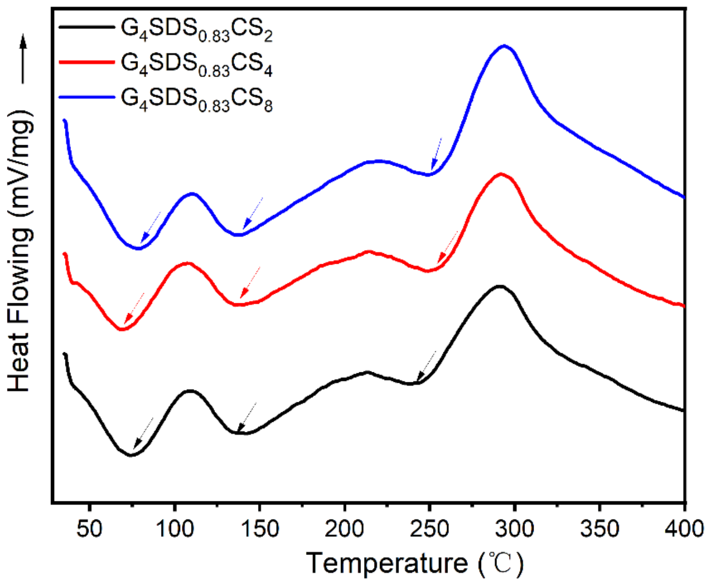

Figure S1. DSC thermograms.of $G_4SDS_{0.83}CS_2$, $G_4SDS_{0.83}CS_4$, and $G_4SDS_{0.83}CS_8$ samples

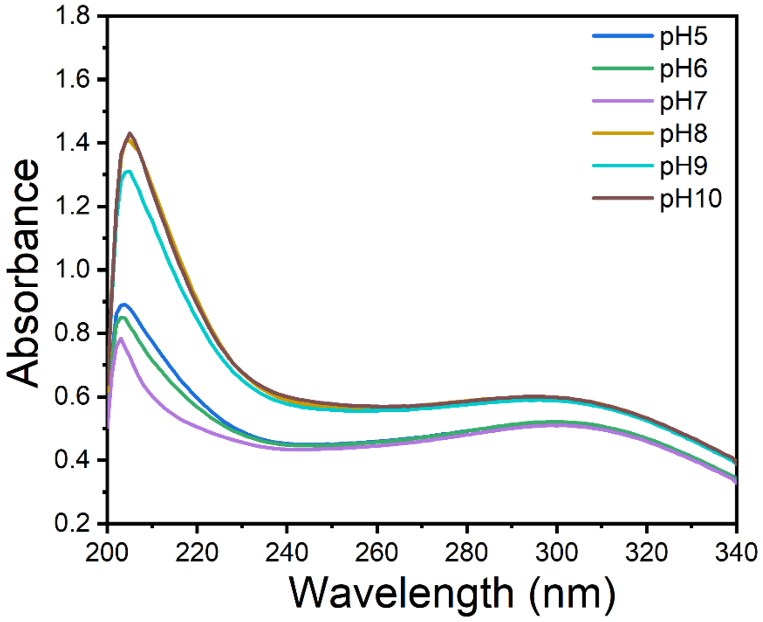

Figure S2. UV spectra of $G_4SDS_{0.83}CS_4$ sample at different pH.

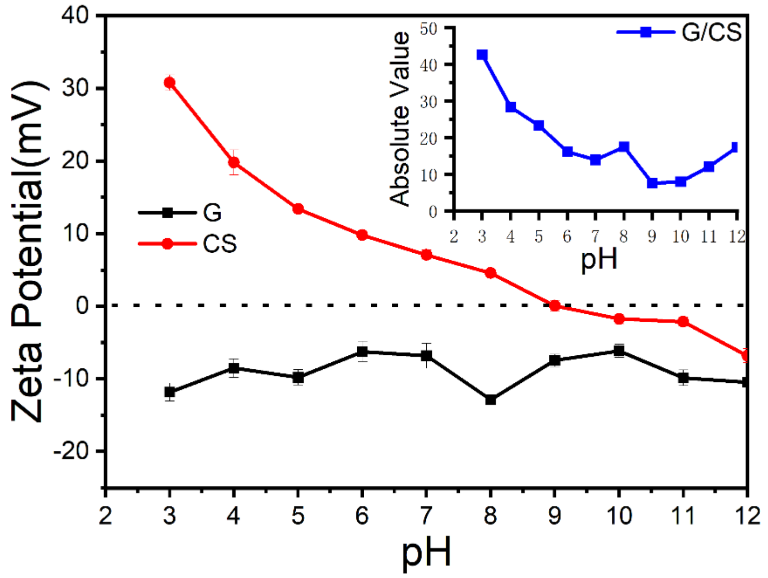

Figure S3. Zeta potential and the absolute potential value of G and CS at different pH.
